# Supplementary material for: Safety Evaluation for Acute and Chronic Oral Toxicity of Maha Pigut Triphala Contains Three Medicinal Fruits in Sprague-Dawley Rats
Source: Biology (Basel). 2024 Dec 2;13(12):1005. doi: 10.3390/biology13121005 (PMC11673089; doi:10.3390/biology13121005)
Supplement: Supplementary file 1 [file biology-13-01005-s001.zip › biology-3305629-supplementary.pdf]

**Table S1.** Hippocratic screening for acute oral toxicity of Triphala formula in female rats

| Triphala formula | Hours after drug administration | | | | | | | | | | | | | | | | | | | | | | | |
|---|---|---|---|---|---|---|---|---|---|---|---|---|---|---|---|---|---|---|---|---|---|---|---|---|
| 5,000 mg/kg | 1 | 2 | 3 | 4 | 5 | 6 | 7 | 8 | 9 | 10 | 11 | 12 | 13 | 14 | 15 | 16 | 17 | 18 | 19 | 20 | 21 | 22 | 23 | 24 |
| Decrease of motor activity | 0 | 0 | 0 | 0 | 0 | 0 | 0 | 0 | 0 | 0 | 0 | 0 | 0 | 0 | 0 | 0 | 0 | 0 | 0 | 0 | 0 | 0 | 0 | 0 |
| Decrease of respiratory rate | 0 | 0 | 0 | 0 | 0 | 0 | 0 | 0 | 0 | 0 | 0 | 0 | 0 | 0 | 0 | 0 | 0 | 0 | 0 | 0 | 0 | 0 | 0 | 0 |
| Loss of righting reflex | 0 | 0 | 0 | 0 | 0 | 0 | 0 | 0 | 0 | 0 | 0 | 0 | 0 | 0 | 0 | 0 | 0 | 0 | 0 | 0 | 0 | 0 | 0 | 0 |
| Loss of screen grip | 0 | 0 | 0 | 0 | 0 | 0 | 0 | 0 | 0 | 0 | 0 | 0 | 0 | 0 | 0 | 0 | 0 | 0 | 0 | 0 | 0 | 0 | 0 | 0 |
| Time of death (hr.) | - | - | - | - | - | - | - | - | - | - | - | - | - | - | - | - | - | - | - | - | - | - | - | - |

Decrease in motor activity: 0 = no decrease in motor activity, no change in respiratory rate, no loss of righting reflex, no loss of screen grip; +1 = does not move spontaneously, but when handled will move rapidly; +2 = when handled will move slowly; +3 = when handled will move sluggishly; +4 = when handled will not move at all. Decrease in respiration rate: +1 = 10% decrease in respiratory rate; +2 = 20% decrease in respiratory rate; +3 = 40% decrease in respiratory rate; +4 = 80% decrease in respiratory rate. Loss of righting reflex: +1 = can be placed only on one side; +2 = can be placed on either side equally well; +3 = can be placed on the back as well as either side; +4 = cannot be aroused from the back position by the hind leg toe pinch. Loss of screen grip: +1 = rat falls off at first shake of the screen; +2 = rat falls off when the screen has been inverted; +3 = rat falls off when the screen is at a 90° angle; +4 = rat falls off as the screen is tilted to a 45° angle.

**Table S2.** Hippocratic screening for chronic oral toxicity of Triphala formula in female and male rats

| **Triphala formula (mg/kg)** | **Weeks after drug administration for female rats** | | | | | | | | | | | | | | | | | | | | |
|---|---|---|---|---|---|---|---|---|---|---|---|---|---|---|---|---|---|---|---|---|---|
| **600, 1,200, 2,400, 2,400 (Satellite)** | 1-2 | 3-4 | 5-6 | 7-8 | 9-10 | 11-12 | 13-14 | 15-16 | 17-18 | 19-20 | 21-22 | 23-24 | 25-26 | 27-28 | 29-30 | 31-32 | 33-34 | 35-36 | 37-38 | 39-40 | 41-42 |
| Decrease of motor activity | 0 | 0 | 0 | 0 | 0 | 0 | 0 | 0 | 0 | 0 | 0 | 0 | 0 | 0 | 0 | 0 | 0 | 0 | 0 | 0 | 0 |
| Decrease of respiratory rate | 0 | 0 | 0 | 0 | 0 | 0 | 0 | 0 | 0 | 0 | 0 | 0 | 0 | 0 | 0 | 0 | 0 | 0 | 0 | 0 | 0 |
| Loss of righting reflex | 0 | 0 | 0 | 0 | 0 | 0 | 0 | 0 | 0 | 0 | 0 | 0 | 0 | 0 | 0 | 0 | 0 | 0 | 0 | 0 | 0 |
| Loss of screen grip | 0 | 0 | 0 | 0 | 0 | 0 | 0 | 0 | 0 | 0 | 0 | 0 | 0 | 0 | 0 | 0 | 0 | 0 | 0 | 0 | 0 |
| Time of death (wk.) | - | - | - | - | - | - | - | - | - | - | - | - | - | - | - | - | - | - | - | - | - |
| **Triphala formula (mg/kg)** | **Weeks after drug administration for male rats** | | | | | | | | | | | | | | | | | | | | |
| **600, 1,200, 2,400, 2,400 (Satellite)** | 1-2 | 3-4 | 5-6 | 7-8 | 9-10 | 11-12 | 13-14 | 15-16 | 17-18 | 19-20 | 21-22 | 23-24 | 25-26 | 27-28 | 29-30 | 31-32 | 33-34 | 35-36 | 37-38 | 39-40 | 41-42 |
| Decrease of motor activity | 0 | 0 | 0 | 0 | 0 | 0 | 0 | 0 | 0 | 0 | 0 | 0 | 0 | 0 | 0 | 0 | 0 | 0 | 0 | 0 | 0 |
| Decrease of respiratory rate | 0 | 0 | 0 | 0 | 0 | 0 | 0 | 0 | 0 | 0 | 0 | 0 | 0 | 0 | 0 | 0 | 0 | 0 | 0 | 0 | 0 |
| Loss of righting reflex | 0 | 0 | 0 | 0 | 0 | 0 | 0 | 0 | 0 | 0 | 0 | 0 | 0 | 0 | 0 | 0 | 0 | 0 | 0 | 0 | 0 |
| Loss of screen grip | 0 | 0 | 0 | 0 | 0 | 0 | 0 | 0 | 0 | 0 | 0 | 0 | 0 | 0 | 0 | 0 | 0 | 0 | 0 | 0 | 0 |
| Time of death (wk.) | - | - | - | - | - | - | - | - | - | - | - | - | - | - | - | - | - | - | - | - | - |

Decrease in motor activity: 0 = no decrease in motor activity, no change in respiratory rate, no loss of righting reflex, no loss of screen grip; +1 = does not move spontaneously, but when handled will move rapidly; +2 = when handled will move slowly; +3 = when handled will move sluggishly; +4 = when handled will not move at all. Decrease in respiration rate: +1 = 10% decrease in respiratory rate; +2 = 20% decrease in respiratory rate; +3 = 40% decrease in respiratory rate; +4 = 80% decrease in respiratory rate. Loss of righting reflex: +1 = can be placed only on one side; +2 = can be placed on either side equally well; +3 = can be placed on the back as well as either side; +4 = cannot be aroused from the back position by the hind leg toe pinch. Loss of screen grip: +1 = rat falls off at first shake of the screen; +2 = rat falls off when the screen has been inverted; +3 = rat falls off when the screen is at a 90° angle; +4 = rat falls off as the screen is tilted to a 45° angle.
